# Near-Infrared Dye-Loaded Thermosensitive Hydrogels as Novel Fluorescence Tissue Markers

**DOI:** 10.3390/gels11080649

**Published:** 2025-08-15

**Authors:** Seon Sook Lee, Yongdoo Choi

**Affiliations:** Division of Technology Convergence, National Cancer Center, 323 Ilsan-ro, Goyang 10408, Republic of Korea; 74752@ncc.re.kr

**Keywords:** surgical marker, laparoscopic surgery, near-infrared fluorescence imaging, thermosensitive polymer

## Abstract

Accurate intraoperative localization of deep-seated lesions remains a major challenge in minimally invasive procedures such as laparoscopic and robotic surgeries. Current marking strategies—including ink tattooing and metallic clips—are limited by dye diffusion, or poor intraoperative visibility. To address these issues, we developed and evaluated four thermosensitive injectable hydrogel systems incorporating indocyanine green-human serum albumin (ICG-HSA) complexes: (1) hexanoyl glycol chitosan (HGC), (2) Pluronic F-127, (3) PCL–PEG–PCL, and (4) PLA–PEG–PLA. All hydrogel formulations exhibited sol–gel transitions at physiological temperatures, facilitating in situ dye entrapment and prolonged fluorescence retention. In vivo fluorescence imaging revealed that HGC and Pluronic F-127 hydrogels retained signals for up to five and two days, respectively. In contrast, polyester-based hydrogels (PCL–PEG–PCL and PLA–PEG–PLA) preserved fluorescence for up to 21–30 days. PLA–PEG–PLA showed the highest signal-to-background ratios and sustained intensity, while PCL–PEG–PCL also achieved long-term retention. These findings suggest that thermosensitive hydrogels incorporating ICG-HSA complexes represent promising tissue marker platforms for real-time, minimally invasive, and long-term fluorescence-guided lesion tracking.

## 1. Introduction

Laparoscopic surgery has become a standard approach across many surgical disciplines, surpassing open surgery due to its minimally invasive nature. This technique offers improved postoperative outcomes, including reduced blood loss, preservation of organ function, faster recovery, and a lower risk of postoperative pain and infection [1]. With the advent of robotic-assisted systems, laparoscopic techniques have continued to evolve and are now considered a cornerstone of modern surgery.

Despite these advances, intraoperative localization of target lesions remains challenging due to limited depth perception and visual field mismatches between endoscopic and laparoscopic views [2]. These limitations can lead to inconsistent incision placement and reduced surgical accuracy.

To address these challenges, various lesion-marking methods have been employed, such as ink tattooing (e.g., methylene blue or India ink) and metallic clip placement [3,4]. However, each method has notable drawbacks: injected dyes may diffuse into surrounding tissues, leading to a significantly enlarged marked area; metal clips are not externally visible and thus are not suitable for laparoscopic procedures [5,6].

In addition, neoadjuvant chemotherapy (NAC) is widely used in the treatment of various solid tumors, including breast, non-small-cell lung, gastric, colorectal, and rectal cancers, to reduce tumor size prior to surgical resection [7,8,9,10]. Although long-term, radiation-free monitoring of tumors during NAC is desirable, no reliable method currently exists. Conventional breast tissue markers consist of polymeric pellets containing radio-opaque metallic components (e.g., metal coils) designed for X-ray imaging [11]. These markers are typically implanted into tumor sites under ultrasound guidance using a 14-gauge needle and are tracked by mammography during NAC. However, such metallic pellet-type markers not only expose surrounding healthy tissues to radiation during X-ray imaging but are also ill-suited for real-time visualization of tumor sites during surgery. Therefore, there is a critical need for a safe, non-invasive, long-lasting, and real-time visible tissue marker capable of accurately localizing deep-seated lesions.

**Figure 1:**
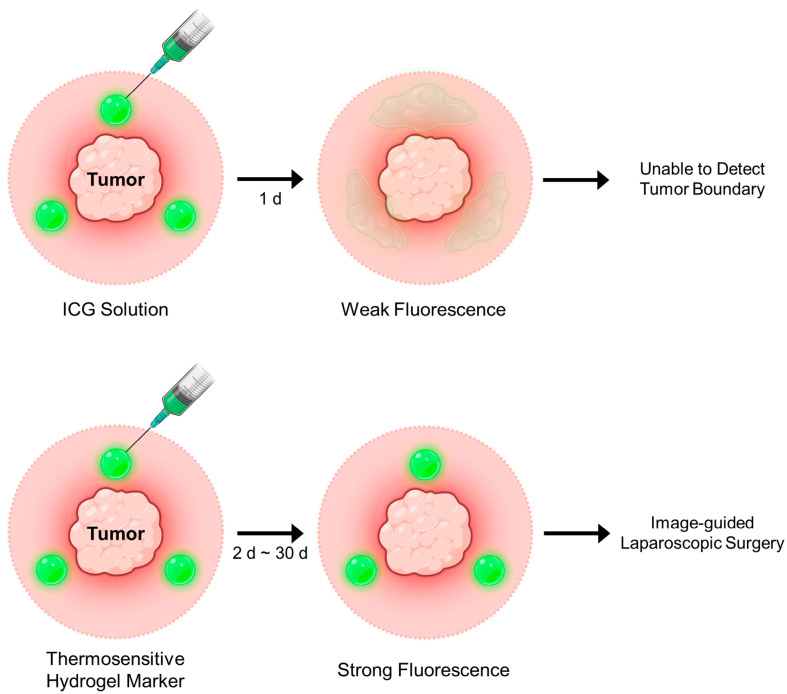
Schematic illustration of fluorescence imaging-guided tumor surgery following marking with a near-infrared dye-loaded thermosensitive hydrogel.

Near-infrared (NIR) fluorescent dyes offer great potential for marking deeply located lesions due to their superior tissue penetration and low background autofluorescence [12]. Among them, indocyanine green (ICG), an FDA-approved NIR dye, is widely used for real-time fluorescence-guided imaging. However, its rapid diffusion and clearance from the injection site limit its utility as a long-lasting fluorescent tissue marker (Figure 1) [13]. We previously developed an ICG-loaded injectable alginate hydrogel for tumor site marking [14]. This system enabled fluorescence labeling of target tissues for up to 96 h post-injection in a mouse model, and the injection sites were clearly visualized in real time during laparoscopic surgery in a porcine model. More recently, polymeric microspheres encapsulating ICG-human serum albumin (HSA) complexes have shown great potential as fluorescent tissue markers for both real-time imaging of tumor sites and long-term tracking for over 30 days [15,16].

In this study, we developed NIR dye-loaded thermosensitive hydrogels as novel fluorescent tissue markers (Figure 1). Aqueous solutions containing thermosensitive polymers and ICG can be easily injected into target lesions using a syringe needle or catheter. Upon injection, the hydrogel undergoes a sol-to-gel transition in situ due to the local temperature increase, resulting in physical entrapment of the dye and prolonged fluorescence signal retention. The marked sites can subsequently be visualized in real time over extended periods, enabling both long-term, noninvasive monitoring of tumor sites and fluorescence imaging-guided surgery.

## 2. Results and Discussion

**Figure 2:**
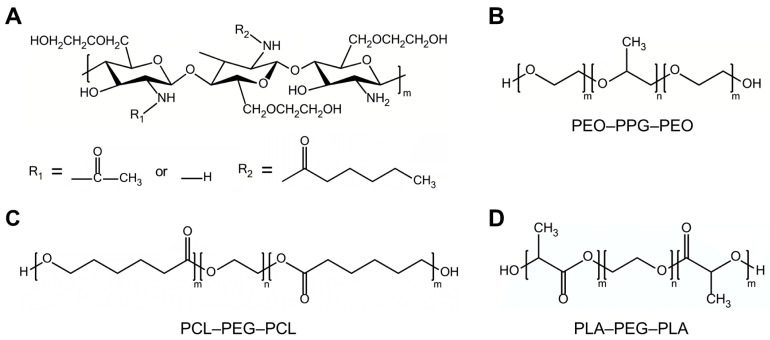
Chemical structures of temperature-sensitive polymers. (**A**) HGC. (**B**) Pluronic F-127 (PEO-PPG-PEO). (**C**) PCL-PEG-PCL. (**D**) PLA-PEG-PLA.

To assess the effect of hydrogel composition on long-term marking, we investigated four thermosensitive hydrogel systems: (1) hexanoyl glycol chitosan (HGC), (2) Pluronic F-127 (F-127), (3) poly(ε-caprolactone)-poly(ethylene glycol)-poly(ε-caprolactone) (PCL–PEG–PCL) triblock copolymer, and (4) poly(D,L-lactide)-b-poly(ethylene glycol)-b-poly(D,L-lactide) (PLA–PEG–PLA) triblock copolymer. As shown in Figure 2, these hydrogels differ in their molecular architectures and amphiphilic block compositions, which influence micelle formation, gelation temperature, and network structure. These structural differences are expected to affect the stability and diffusion of encapsulated NIR dyes in vivo.

### 2.1. Evaluation of the Thermogelling Properties and Dye Retention of the HGC Hydrogel

Chitosan, a naturally derived and non-sulfated glycosaminoglycan, is a hydrophilic, biocompatible, and biodegradable polymer [17]. However, it has low mechanical strength and is only soluble under acidic conditions. To overcome these limitations, glycol groups were introduced to enhance solubility at neutral pH, and thermosensitivity was achieved by grafting hexanoyl groups onto the hydrophilic backbone, resulting in the formation of HGC [18]. It is well known that, as the temperature increases, hydrophobic interactions among these moieties are enhanced, leading to the formation of a physical network and a transition to a non-flowing gel state [19].

**Figure 3:**
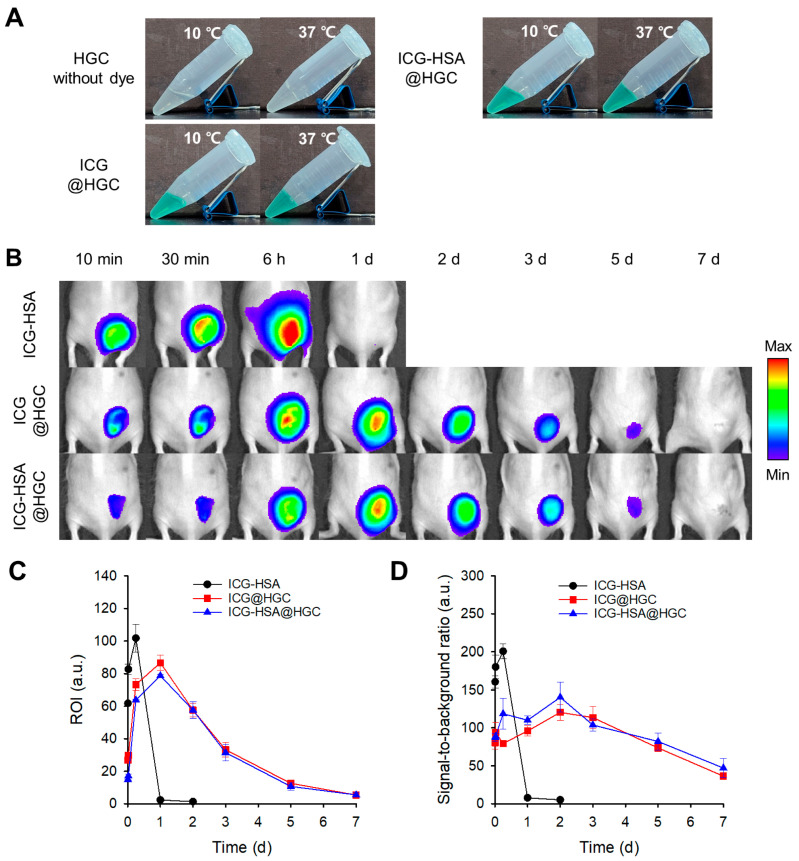
(**A**) Sol–gel transition images of HGC and HGC samples loaded with ICG (ICG@HGC) or ICG-HSA (ICG-HSA@HGC) at 10 °C and 37 °C, respectively. (**B**) In vivo evaluation of ICG-HSA solution and HGC thermogels in mice. NIR fluorescence images (λ_ex_. = 780 nm, λ_em_. = 845 nm) were obtained at defined time intervals. Changes in (**C**) region of interest (ROI) fluorescence intensity and (**D**) signal-to-background ratio (SBR) at defined time intervals.

The HGC hydrogel, prepared at 4 *w*/*v*%, exhibited thermoresponsive sol–gel transition behavior: it remained in a sol state at 10 °C and transitioned to a gel at 37 °C when loaded with either ICG or ICG-HSA (Figure 3A). Notably, the HGC gel exhibited sol–gel transition even at relatively low polymer concentrations (3–7 *w*/*v*%), as previously reported [20].

To evaluate in vivo efficacy of the samples for NIR fluorescence marking of the target site, sample solutions (0.1 mL each) were subcutaneously injected into the dorsal surface of SKH-1 hairless mice. As expected, in the ICG-HSA injection group, which did not contain hydrogel polymer, fluorescence signals rapidly diffused from the injection site into the surrounding tissues within 6 h post-injection, indicating easy dispersion of the injected ICG-HSA complex. The fluorescence signals at the injection site had mostly disappeared by 24 h post-injection (Figure 3B). In contrast, when HGC hydrogels loaded with ICG (ICG@HGC) or ICG-HSA (ICG-HSA@HGC) were injected for fluorescence marking, fluorescence signals were retained at the injection site for at least five days, and dye diffusion into the surrounding tissues was markedly reduced compared to the ICG-HSA group. Quantitative analysis revealed that 20% and 37% of the initial fluorescence signals remained after seven days in the ICG@HGC and ICG-HSA@HGC groups, respectively (Figure 3C), with significantly high signal-to-background ratios (SBRs) of 36 and 47 (Figure 3D).

This improved retention is attributed to the network structure of HGC, driven by hydrophobic interactions among hexanoyl side chains introduced through chemical modification of glycol chitosan [19]. However, the encapsulated dye gradually diffused into the surrounding tissue, limiting signal retention to approximately one week. These findings suggest that HGC hydrogels provide a modest improvement over the free dye formulation (i.e., ICG-HSA), but their utility may be limited in applications requiring prolonged signal retention over multiple weeks.

### 2.2. Evaluation of the Thermogelling Properties and Dye Retention of the F-127 Hydrogel

F-127 is an FDA-approved, amphiphilic ABA-type triblock copolymer composed of hydrophilic poly(ethylene oxide) (PEO) chains at both ends and a hydrophobic poly(propylene oxide) (PPO) core [21,22]. Owing to its thermoresponsive behavior and biocompatibility, it has been extensively utilized in drug delivery applications [23,24]. In aqueous solution at low temperatures, individual polymer chains remain dispersed due to the hydration of the PEO segments. However, as the temperature increases, the PPO blocks become increasingly hydrophobic due to reduced hydrogen bonding with surrounding water molecules [25]. This temperature-induced hydrophobicity promotes micelle formation, wherein the PPO segments aggregate to form hydrophobic cores, while the PEO chains extend outward into the aqueous environment. At sufficiently high concentrations, further temperature elevation induces micelle packing and entanglement, resulting in gel formation. This sol-to-gel transition is reversible and highly sensitive to both polymer concentration and ambient temperature.

**Figure 4:**
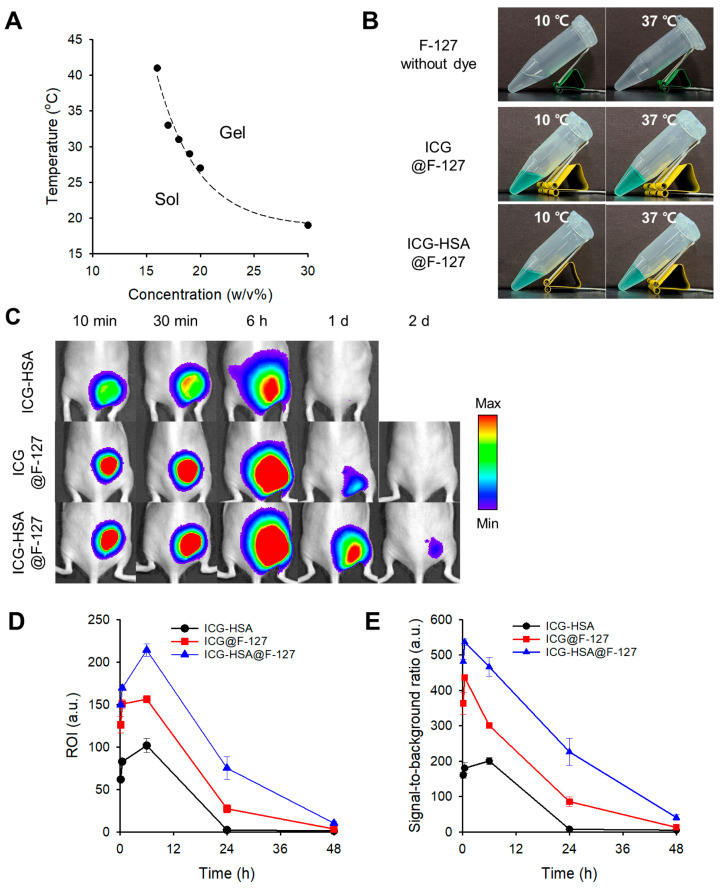
(**A**) Sol–gel transition diagram of F-127 solutions at various concentrations. (**B**) Sol–gel transition images of F-127 hydrogels loaded with ICG (ICG@F-127) or ICG-HSA (ICG-HSA@F-127) at 10 °C and 37 °C. (**C**) In vivo evaluation of ICG-HSA solution and F-127 thermogels in mice. NIR fluorescence images (λ_ex_. = 780 nm, λ_em_. = 845 nm) were obtained at defined time intervals. (**D**,**E**) Quantitative analysis of NIR fluorescence images. (**D**) Fluorescence intensity in the ROI. (**E**) SBR at defined time intervals.

In this study, sol–gel transitions were evaluated across various concentrations (16–30 *w*/*v*%), with transition temperatures ranging from 41 °C to 19 °C (Figure 4A). Based on these results, an 18 *w*/*v*% F-127 solution, exhibiting a transition temperature of 31 °C, was selected for further investigation. This formulation remained in the sol state at 10 °C and formed a gel at 37 °C upon loading with ICG or ICG-HSA (Figure 4B).

For the in vivo efficacy test, F-127 hydrogels loaded with ICG (ICG@F-127) or ICG-HSA (ICG-HSA@F-127) were injected into mice as described above. Bright fluorescence signals at the injection sites were retained for up to two days in both groups (Figure 4C). Notably, the ICG-HSA@F-127 group exhibited higher fluorescence intensity and SBR values than the ICG@F-127 group. At 24 h post-injection, fluorescence intensities remained at 22% (ICG@F-127) and 50% (ICG-HSA@F-127), with corresponding SBRs of 86 and 226, respectively (Figure 4D,E).

Although fluorescence signals at the injection sites were more prolonged compared to the ICG-HSA solution group, the area of fluorescence emission similarly expanded into surrounding tissues within 6 h post-injection. These findings indicate that, despite improved fluorescence signal intensity and short-term retention, the F-127 hydrogel formulation is not highly effective for accurate and long-term fluorescent marking.

This limitation may stem from the relatively poor mechanical stability of F-127 in vivo. Under physiological conditions, it is susceptible to dilution and erosion due to osmotic effects and the presence of interstitial fluids [26], leading to the gradual disassembly of the micellar network and subsequent diffusion of the encapsulated dye into surrounding tissues. Despite the advantage of FDA approval, F-127 is inherently limited in applications requiring sustained fluorescence retention over extended periods.

### 2.3. Evaluation of the Thermogelling Properties and Dye Retention of the PCL-PEG-PCL and PLA-PEG-PLA Hydrogels

PCL-PEG-PCL and PLA-PEG-PLA, both of which possess an amphiphilic triblock copolymer architecture, were also investigated for their fluorescence-preserving capabilities. PCL and PLA, two well-known biodegradable polyesters, were selected due to their excellent biocompatibility [27]. In these copolymers, the central hydrophilic block consists of PEG, while the terminal hydrophobic blocks are composed of PCL or PLA, respectively (Figure 2). When dissolved in an aqueous environment, these amphiphilic copolymers self-assemble into flower-like micelles, in which the hydrophobic segments form the core and the PEG chains extend outward [28]. Upon temperature increase, hydrophobic interactions among the terminal blocks are strengthened, promoting micelle aggregation and subsequent gel formation.

**Figure 5:**
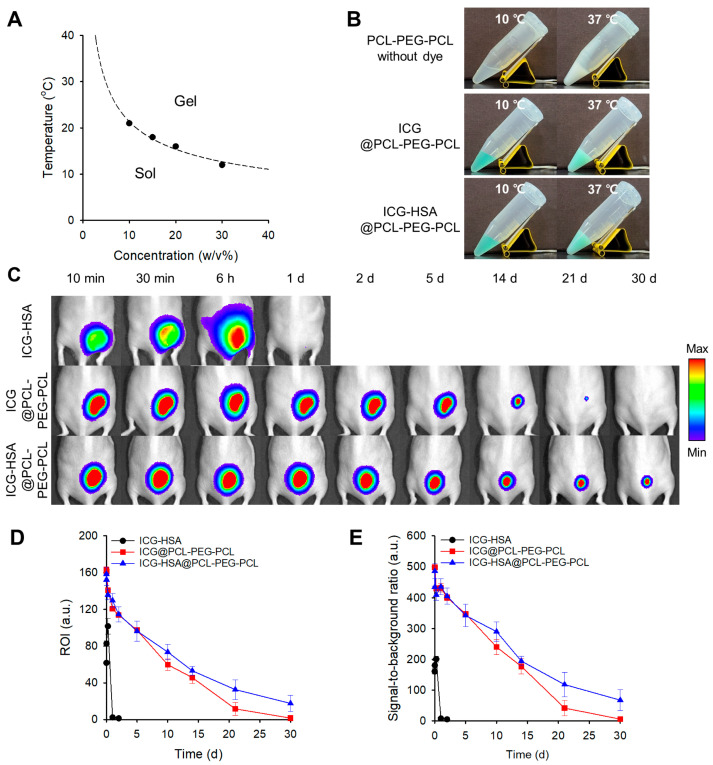
(**A**) Sol–gel transition diagram of PCL-PEG-PCL solutions at various concentrations. (**B**) Sol–gel transition images of PCL-PEG-PCL hydrogels loaded with ICG (ICG@PCL-PEG-PCL) or ICG-HSA (ICG-HSA@PCL-PEG-PCL) at 10 °C and 37 °C. (**C**) In vivo evaluation of ICG-HSA solution and PCL-PEG-PCL thermogels in mice. NIR fluorescence images (λ_ex_. = 780 nm, λ_em_. = 845 nm) were acquired at defined time intervals. (**D**,**E**) Quantitative analysis of NIR fluorescence images. (**D**) Fluorescence intensity in the ROI. (**E**) SBR at defined time intervals.

PCL-PEG-PCL solutions exhibited concentration-dependent sol–gel transitions, with transition temperatures ranging from 21 °C to 12 °C for 10–30 *w*/*v*% solutions (Figure 5A). A 10 *w*/*v*% formulation, exhibiting a transition temperature of 21 °C, was selected for further investigation. Gelation of 10 *w*/*v*% PCL-PEG-PCL hydrogels loaded with either ICG (ICG@PCL-PEG-PCL) or ICG-HSA (ICG-HSA@PCL-PEG-PCL) was confirmed at 37 °C (Figure 5B).

Upon subcutaneous injection of ICG@PCL-PEG-PCL into mice, fluorescence signals persisted for up to 30 days (Figure 5C,D). Interestingly, in the ICG-HSA@PCL-PEG-PCL group, 11% of the initial signal remained even at 30 days post-injection. SBRs of 6 and 67 were measured on day 30 for ICG@PCL-PEG-PCL and ICG-HSA@PCL-PEG-PCL, respectively (Figure 5E). Notably, no significant expansion of the fluorescence signal area at the injection sites was observed throughout the test period, which is a critical feature for precision imaging-guided surgery.

**Figure 6:**
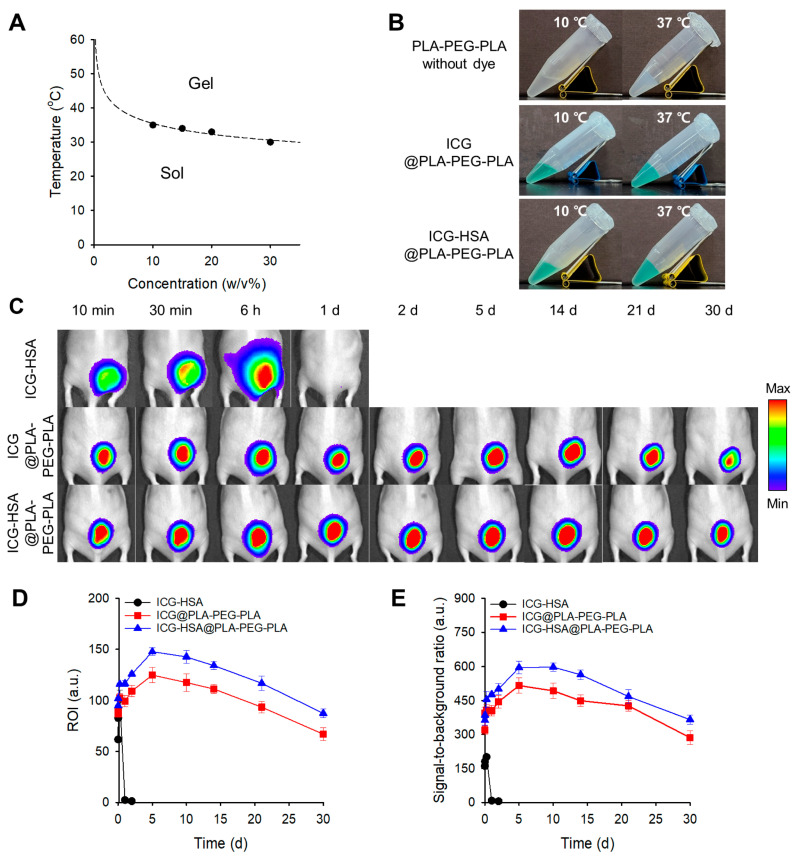
(**A**) Sol–gel transition diagram of PLA-PEG-PLA solutions at various concentrations. (**B**) Sol–gel transition images of PLA-PEG-PLA hydrogels loaded with ICG (ICG@ PLA-PEG-PLA) or ICG-HSA (ICG-HSA@ PLA-PEG-PLA) at 10 °C and 37 °C. (**C**) In vivo evaluation of ICG-HSA solution and PLA-PEG-PLA thermogels in mice. NIR fluorescence images (λ_ex_. = 780 nm, λ_em_. = 845 nm) were acquired at defined time intervals. (**D**,**E**) Quantitative analysis of NIR fluorescence images. (**D**) Fluorescence intensity in the ROI. (**E**) SBR at defined time intervals.

Next, PLA-PEG-PLA solutions exhibited sol–gel transitions in the temperature range of 35–30 °C, depending on the concentration (10–30 *w*/*v*%). A 30 *w*/*v*% formulation, with a transition temperature of 30 °C, was selected for further investigation (Figure 6A). Gelation of 30 *w*/*v*% PLA-PEG-PLA hydrogels loaded with either ICG (ICG@PLA-PEG-PLA) or ICG-HSA (ICG-HSA@PLA-PEG-PLA) was also confirmed at 37 °C (Figure 6B).

In vivo NIR fluorescence imaging using the PLA-PEG-PLA hydrogel formulation demonstrated the most favorable performance as a fluorescent tissue marker (Figure 6). While the ICG-HSA@PLA-PEG-PLA group showed higher ROI and SBR values compared to the ICG@PLA-PEG-PLA group, both ICG- and ICG-HSA-loaded PLA-PEG-PLA hydrogels exhibited strong fluorescence retention throughout the 30-day study period (Figure 6C), maintaining 77% and 92% of the initial signal with outstanding SBRs of 286 and 365 on day 30, respectively (Figure 6D,E).

The fluorescence signal area at the injection sites remained stable over time, indicating that the encapsulated ICG dyes were effectively retained within the hydrogels and that no significant diffusion into surrounding tissues occurred.

The long-lasting fluorescence observed in the PCL-PEG-PCL and PLA-PEG-PLA hydrogel formulations is likely attributed to the stable micellar structures and intermicellar bridging among the terminal hydrophobic blocks (PCL or PLA), which lead to denser gel networks that effectively restrict dye diffusion [29].

Previous studies have reported that PCL–PEG–PCL (PCEC, 1000–1000–1000 Da) undergoes sol–gel transition at a lower temperature than PEG–PCL–PEG (PECE, 550–2000–550 Da), despite having comparable molecular weights and PEG/PCL ratios. This difference has been attributed to the topological advantage of PCEC, which enables micelle bridging—a feature absent in PECE [30].

In addition, PCL–PEG–PCL hydrogels exhibit significantly higher mechanical strength (G′ ≈ 10,000 Pa) compared to PEG–PCL–PEG gels (G′ ≈ 100 Pa), owing to enhanced micelle stability and more compact network formation [31].

Some sterilization methods (e.g., gamma irradiation and ethylene oxide [EO] gas) can alter the molecular weight and physicochemical properties of biopolymers [16,32]. Therefore, selecting an appropriate sterilization method is crucial for the clinical application of hydrogel-based fluorescent tissue markers.

In addition, the choice of needle or injector for hydrogel solution delivery should depend on the target site within the human body. For instance, breast tissue markers are typically administered through 17- or 18-gauge needles to mark breast cancer lesions [33], which makes hydrogel injection relatively straightforward. In contrast, when the target site is the submucosal space of the stomach, 21–25 G needles equipped with a 1.8-m-long injection catheter are generally used [34]. In such cases, considerably greater force is required for injection. Therefore, the injectability of the hydrogel solution should be carefully evaluated during the design of hydrogel markers for clinical applications.

## 3. Conclusions

In this study, we developed and evaluated four injectable thermosensitive hydrogels to enhance the retention of NIR fluorescent dyes at target tissues. All hydrogels underwent sol–gel transition at physiological temperature and enabled localized retention of NIR fluorescence signals following subcutaneous injection.

While F-127 and HGC exhibited limited fluorescence duration, the BAB-type triblock copolymers PLA–PEG–PLA and PCL–PEG–PCL demonstrated significantly prolonged fluorescence retention of up to 30 days. Notably, the PLA–PEG–PLA hydrogel formulation achieved the highest fluorescence intensity at the injection site and exhibited excellent long-term performance. The architectural advantage of the hydrophobic end blocks in these triblock copolymers facilitated micelle formation and intermicellar bridging, resulting in stable in situ gels with high signal localization. Since each hydrogel retains fluorescence signals for a different duration, selection of the appropriate hydrogel should be made according to the intended application.

Overall, these injectable thermosensitive hydrogel markers undergo sol–gel transition at body temperature, enable controlled dye retention, and can be tailored to meet diverse clinical needs based on their degradation and retention profiles. This platform provides a versatile and biocompatible strategy for tissue marking and fluorescence imaging-guided surgery, particularly in procedures requiring long-term lesion visualization.

## 4. Materials and Methods

### 4.1. Materials

HGC (LabThermogel) was obtained from LabToLab Co., Ltd. (Daejeon, Republic of Korea). F-127 was purchased from Sigma-Aldrich (St. Louis, MO, USA). PCL-PEG-PCL (MW 1000-1000-1000 Da) and PLA-PEG-PLA (MW 1500-1500-1500 Da) were purchased from Akina Inc. (West Lafayette, IN, USA). Indocyanine green (ICG, Diagnogreen) was purchased from Daiichi Sankyo Co., Ltd. (Tokyo, Japan), and human serum albumin (HSA, SK Albumin 20% Inj.) was obtained from SK Plasma Co., Ltd. (Seongnam, Republic of Korea).

### 4.2. Phase Diagram of Thermosensitive Hydrogels

To determine the sol–gel transition temperature of each polymer, aqueous solutions of various concentrations were prepared and subjected to heating gradually. HGC was used at a concentration of 4 *w*/*v*% according to the manufacturer’s instruction.

A total of 2 mL of deionized water was added to 0.2–0.6 g of F-127, PCL-PEG-PCL, or PLA-PEG-PLA, and the mixture was stirred at 4 °C to prepare 10–30 *w*/*v*% polymer solutions. For phase transition analysis, 0.5 mL of each polymer solution was transferred into the Eppendorf tubes and heated gradually from 10 to 45 °C using a heating block (ThermoMixer C, Eppendorf, Germany). The temperature was increased in 1 °C increments, with a stabilization time of 5 min at each step. The sol–gel transition temperature was determined using the tube inversion method, with gelation defined as the point at which the solution no longer flowed upon inversion. The tube containing the hydrogel solution was inverted, and the flow of the solution was observed for 3 s. If the sample retained its shape even when the tube was gently shaken from side to side, it was classified as a gel. Samples that exhibited any noticeable flow were classified as a sol.

### 4.3. Gelation Behavior with Fluorescent Dyes

To assess the gelation behavior in the presence of fluorescent dyes, ICG or ICG-HSA complexes were mixed with each thermosensitive polymer at the following gel-forming concentrations: HGC (4 *w*/*v*%), F-127 (18 *w*/*v*%), PCL-PEG-PCL (10 *w*/*v*%), and PLA-PEG-PLA (30 *w*/*v*%). HGC was used at 4 *w*/*v*%, based on its reported sol–gel transition temperature of 32 °C.

Each polymer was pre-dissolved at 4 °C. For ICG loading, 0.5 mg of ICG was dissolved in 0.6 mL of deionized water, and 0.12 mL of this solution was added to the polymer solution. For ICG-HSA preparation, 0.5 mg of ICG was dissolved in 0.4 mL of deionized water and mixed with 0.2 mL of HSA solution. Then, 0.12 mL of the ICG-HSA complex was added to the polymer solution. In all samples, the final concentration of ICG and HSA were adjusted to 30 μM.

Aliquots (0.5 mL) of the dye-loaded polymer solutions were transferred to Eppendorf tubes. Each sample was incubated for 5 min at 10 °C and then at 37 °C using a heating block, followed by assessment of gelation via the tube inversion method.

### 4.4. In Vivo Evaluation of Dye-Loaded Thermosensitive Hydrogel Markers in a Mouse Model

To evaluate the efficacy of the dye-loaded thermosensitive hydrogels as fluorescent tissue markers, the retention and diffusion of the dyes at the injection site were observed in vivo using a mouse model. All animal procedures were approved by the Institutional Animal Care and Use Committee of the National Cancer Center Research Institute (approval No. NCC-23-915, NCC-24-1009) of Republic of Korea. SKH-1 hairless mice (5 weeks old, male) were obtained from Orient Bio (Seoul, Republic of Korea).

Mice were divided into three groups (*n* = 3 per group): (1) ICG-HSA solution, (2) ICG-loaded thermogels, and (3) ICG-HSA-loaded thermogels. The ICG-HSA solution (30 μM) was prepared in deionized water. Thermogel formulations were prepared by dissolving polymers at 4 °C, then mixing with ICG or ICG-HSA to a final dye concentration of 30 μM. Mice were subcutaneously injected with 0.1 mL of the assigned formulation in the dorsal region.

Fluorescent signals were monitored for up to 30 days using the IVIS Lumina XR system (Xenogen Corporation-Caliper, CA, USA). For quantitative analysis, the NIR fluorescence (excitation = 780 ± 20 nm; emission = 845 ± 40 nm) was obtained using imaging software (Living Image^®^ 2.60.1). The signal-to-background ratio (SBR) was calculated by dividing the region of interest (ROI) fluorescence intensity at the injection site by the background ROI intensity: SBR = ROI (target)/ROI (background).

## Data Availability

The original contributions presented in this study are included in the article. Further inquiries can be directed to the corresponding authors.

## Abbreviations

The following abbreviations are used in this manuscript:

F-127	Pluronic F-127
HGC	Hexanoyl glycol chitosan
HSA	Human serum albumin
ICG	Indocyanine green
LCST	Lower critical solution temperature
NAC	Neoadjuvant chemotherapy
NIR	Near-infrared
PCL	Poly(ε-caprolactone)
PEG	Poly(ethylene glycol)
PEO	Poly(ethylene oxide)
PGA	Poly(glycolide)
PLA	Poly(D,L-lactide)
PPO	Poly(propylene oxide)
ROI	Region of interest
SBR	Signal-to-background ratio

## References

[1] Madhok B., Nanayakkara K., Mahawar K. (2022). Safety considerations in laparoscopic surgery: A narrative review. World J. Gastrointest. Endosec..

[2] Wong S.W., Crowe P. (2023). Visualisation ergonomics and robotic surgery. J. Rob. Surg..

[3] Nowak N., Dziedzic J., Nurczyk K., Zakościelny A., Bury P., Zgodziński W., Zinkiewicz K. (2020). Application of endoscopic tattooing in intraoperative localization of colon tumours and sentinel lymph nodes. J. Pre-Clin. Clin. Res..

[4] Park D.H., Moon H.S., Sul J.Y., Kwon I.S., Yun G.Y., Lee S.H., Park J.H., Kim J.S., Kang S.H., Lee E.S. (2018). Role of preoperative endoscopic clipping in laparoscopic distal gastrectomy for early gastric cancer. Medicine.

[5] Satoyoshi T., Okita K., Ishii M., Hamabe A., Usui A., Akizuki E., Okuya K., Nishidate T., Yamano H., Nakase H. (2021). Timing of indocyanine green injection prior to laparoscopic colorectal surgery for tumor localization: A prospective case series. Surg. Endosc..

[6] Haim Zada M., Gallimidi Z., Schlesinger Laufer M., Nyska A., Domb A.J. (2020). Biodegradable breast tissue marker clip. ACS Appl. Bio Mater..

[7] Korde L.A., Somerfield M.R., Carey L.A., Crews J.R., Denduluri N., Hwang E.S., Khan S.A., Loibl S., Morris E.A., Perez A. (2021). Neoadjuvant chemotherapy, endocrine therapy, and targeted therapy for breast cancer: ASCO guideline. J. Clin. Oncol..

[8] Saw S.P.L., Ong B.H., Chua K.L.M., Takano A., Tan D.S.W. (2021). Revisiting neoadjuvant therapy in non-small-cell lung cancer. Lancet Oncol..

[9] Ling Q., Huang S.T., Yu T.H., Liu H.L., Zhao L.Y., Chen X.L., Liu K., Chen X.Z., Yang K., Hu J.K. (2023). Optimal timing of surgery for gastric cancer after neoadjuvant chemotherapy: A systematic review and meta-analysis. World J. Surg. Oncol..

[10] Gosavi R., Chia C., Michael M., Heriot A.G., Warrier S.K., Kong J.C. (2021). Neoadjuvant chemotherapy in locally advanced colon cancer: A systematic review and meta-analysis. Int. J. Colorectal Dis..

[11] Shah A.D., Mehta A.K., Talati N., Brem R., Margolies L.R. (2019). Reprint of: Breast tissue markers: Why? What’s out there? How do I choose?. Clin. Imaging.

[12] Ullah Z., Roy S., Muhammad S., Yu C., Huang H., Chen D., Long H., Yang X., Du X., Guo B. (2024). Fluorescence imaging-guided surgery: Current status and future directions. Biomater. Sci..

[13] Barberio M., Pizzicannella M., Spota A., Ashoka A.H., Agnus V., Al Taher M., Jansen-Winkeln B., Gockel I., Marescaux J., Swanström L. (2021). Preoperative endoscopic marking of the gastrointestinal tract using fluorescence imaging: Submucosal indocyanine green tattooing versus a novel fluorescent over-the-scope clip in a survival experimental study. Surg. Endosc..

[14] Lee S.S., Kim H., Sohn D.K., Eom J.B., Seo Y.S., Yoon H.M., Choi Y. (2020). Indocyanine green-loaded injectable alginate hydrogel as a marker for precision cancer surgery. Quant. Imaging Med. Surg..

[15] Kim H.J., Choi Y. (2024). Indocyanine green-loaded microspheres as a near-infrared fluorescence marker for long-term localization of tumor sites. J. Pharm. Investig..

[16] Kim H.J., Lee S.S., Sohn D.K., Yoon H.M., Park K.L., Park S.J., Choi Y. (2024). Multiporous PMMA microballs as a novel fluorescence tissue marker. Chem. Eng. J..

[17] Gupta P., Sharma S., Jabin S., Jadoun S. (2024). Chitosan nanocomposite for tissue engineering and regenerative medicine: A review. Int. J. Biol. Macromol..

[18] Yu Y., Kim D.H., Suh E.Y., Jeong S.H., Kwon H.C., Le T.P., Kim Y., Shin S.A., Park Y.H., Huh K.M. (2022). Injectable glycol chitosan thermogel formulation for efficient inner ear drug delivery. Carbohydr. Polym..

[19] Cho I.S., Park C.G., Huh B.K., Cho M.O., Khatun Z., Li Z., Kang S.W., Choy Y.B., Huh K.M. (2016). Thermosensitive hexanoyl glycol chitosan-based ocular delivery system for glaucoma therapy. Acta Biomater..

[20] Li Z., Shim H., Cho M.O., Cho I.S., Lee J.H., Kang S.W., Kwon B., Huh K.M. (2018). Thermo-sensitive injectable glycol chitosan-based hydrogel for treatment of degenerative disc disease. Carbohydr. Polym..

[21] Marques A.C., Costa P.C., Velho S., Amaral M.H. (2023). Injectable poloxamer hydrogels for local cancer therapy. Gels.

[22] Tuszynska M., Skopinska-Wisniewska J., Bartniak M., Bajek A. (2025). Conceptualization and preliminary characterization of poloxamer-based hydrogels for biomedical applications. Bioconjug. Chem..

[23] Lupu A., Bercea M., Avadanei M., Gradinaru L.M., Nita L.E., Gradinaru V.R. (2025). Temperature sensitive pluronic F127-based gels incorporating natural therapeutic agents. Macromol. Mater. Eng..

[24] Khaliq N.U., Lee J., Kim S., Sung D., Kim H. (2023). Pluronic F-68 and F-127 based nanomedicines for advancing combination cancer therapy. Pharmaceutics.

[25] Di Spirito N.A., Grizzuti N., Lutz-Bueno V., Urciuoli G., Auriemma F., Pasquino R. (2024). Pluronic F68 micelles as carriers for an anti-inflammatory drug: A rheological and scattering investigation. Langmuir.

[26] Lupu A., Gradinaru L.M., Rusu D., Bercea M. (2023). Self-healing of pluronic F127 hydrogels in the presence of various polysaccharides. Gels.

[27] Min K.E., Jang J.W., Kim C., Yi S. (2024). Enhancement of mechanical properties of PCL/PLA/DMSO_2_ composites for bone tissue engineering. Appl. Sci..

[28] Lin Q., Lim J.Y.C., Xue K., Chee C.P.T., Loh X.J. (2020). Supramolecular thermogels from branched PCL-containing polyurethanes. RSC Adv..

[29] Dabbaghi A., Ramazani A., Farshchi N., Rezaei A., Bodaghi A., Rezayati S. (2021). Synthesis, physical and mechanical properties of amphiphilic hydrogels based on polycaprolactone and polyethylene glycol for bioapplications: A review. J. Ind. Eng. Chem..

[30] Gong C., Shi S., Wu L., Gou M., Yin Q., Guo Q., Dong P., Zhang F., Luo F., Zhao X. (2009). Biodegradable in situ gel-forming controlled drug delivery system based on thermosensitive PCL-PEG-PCL hydrogel. Part 2: Sol-gel-sol transition and drug delivery behavior. Acta Biomater..

[31] Huynh C.T., Nguyen K., Lee D.S. (2011). Injectable block copolymer hydrogels: Achievements and future challenges for biomedical applications. Macromolecules.

[32] Bhatnagar D., Dube K., Damodaran V.B., Subramanian G., Aston K., Halperin F., Mao M., Pricer K., Murthy N.S., Kohn J. (2016). Effects of terminal sterilization on PEG-based bioresorbable polymers used in biomedical applications. Macromol. Mater. Eng..

[33] UltraCor™ Twirl™ Breast Tissue Marker. https://www.bd.com/en-us/products-and-solutions/products/product-families/ultracor-twirl-breast-tissue-marker.

[34] Clear-Jet Injection Catheter. https://finemedix.com/en/clear-jet-injection-catheter/.

